# Living in Chicago during the Fair

**Published:** 1893-08

**Authors:** J. N. Crouse

**Affiliations:** Chicago


### Domestic Correspondence.

### LIVING IN CHICAGO DURING THE FAIR.

Chicago, July 7, 1893.

Editor of International Dental Journal:

Sir,—From letters received from dentists in different parts of the country I am inclined to think there is a lack of information as to the expense of living in Chicago during the season of the World's Fair. Board and lodging were never more reasonable than now. Rooms can be had, where two are willing to room together, for fifty cents a day each. First-class rooms and accommodations can be had from a dollar to a dollar and a half per day when the parties wish to room alone. The highest-priced hotels are entertaining people for five dollars per day, room and board. For twenty cents, in restaurants just outside of the Fair grounds, will be furnished three eggs, a cup of tea or coffee, and all the bread and butter one wants. For thirty-five cents can be had a good, well-cooked steak, potatoes, a cup of tea or coffee, and bread and butter.

The boat, railway, and street-car companies are doing all in their power to furnish the best and cheapest transportation possible.

Of course there are a few catch-penny schemes, but they are not in connection with the responsible hotels or the Fair. Considering the size of the Exposition, the number is remarkably small.

I have made this investigation for the purpose of informing the dental profession of the exact facts concerning the expense here, and would urge upon every one to remain as long in Chicago as possible. A month or six weeks can be spent very profitably in seeing what the world has done and is doing, and a longer time could be used to great advantage.

J. N. Crouse.

